# Cardioprotective Effect against Ischemia–Reperfusion Injury of PAK-200, a Dihydropyridine Analog with an Inhibitory Effect on Cl^−^ but Not Ca^2+^ Current

**DOI:** 10.3390/biom13121719

**Published:** 2023-11-29

**Authors:** Iyuki Namekata, Miku Tamura, Jyunya Kase, Shogo Hamaguchi, Hikaru Tanaka

**Affiliations:** Department of Pharmacology, Faculty of Pharmaceutical Sciences, Toho University, 2-2-1 Miyama Funabashi, Chiba 274-8510, Japan; iyuki@phar.toho-u.ac.jp (I.N.); 19m3007@g.iuhw.ac.jp (M.T.); thdykbt+jk@gmail.com (J.K.); shogo.hamaguchi@phar.toho-u.ac.jp (S.H.)

**Keywords:** cardioprotection, dihydropyridine, PAK-200, chloride current, ischemia and reperfusion

## Abstract

We examined the effects of a dihydropyridine analog, PAK-200, on guinea pig myocardium during experimental ischemia and reperfusion. In isolated ventricular cardiomyocytes, PAK-200 (1 μM) had no effect on the basal peak inward and steady-state currents but inhibited the isoprenaline-induced time-independent Cl^−^ current. In the right atria, PAK-200 had no effect on the beating rate and the chronotropic response to isoprenaline. In an ischemia–reperfusion model with coronary-perfused right ventricular tissue, a decrease in contractile force and a rise in tension were observed during a period of 30-min no-flow ischemia. Upon reperfusion, contractile force returned to less than 50% of preischemic values. PAK-200 had no effect on the decline in contractile force during the no-flow ischemia but reduced the rise in resting tension. PAK-200 significantly improved the recovery of contractile force after reperfusion to about 70% of the preischemic value. PAK-200 was also shown to attenuate the decrease in tissue ATP during ischemia. Treatment of ventricular myocytes with an ischemia-mimetic solution resulted in depolarization of the mitochondrial membrane potential and an increase in cytoplasmic and mitochondrial Ca^2+^ concentrations. PAK-200 significantly delayed these changes. Thus, PAK-200 inhibits the cAMP-activated chloride current in cardiac muscle and may have protective effects against ischemia–reperfusion injury through novel mechanisms.

## 1. Introduction

The contractile function of the myocardium is controlled by the movement of cations, Na^+^, Ca^2+^, and K^+^ [1]. The transsarcolemmal influx of Na^+^ forms the rapid upstroke of the action potential in the working myocardium and is responsible for the conduction of excitation. The influx of Ca^2+^ during the plateau phase triggers Ca^2+^-induced Ca^2+^ release (CICR) from the sarcoplasmic reticulum (SR) and the resulting rise in cytoplasmic Ca^2+^ concentration causes contraction. Potassium channels are responsible for repolarization and maintenance of the resting membrane potential. The function of these ion channels as well as the contractile proteins are ultimately dependent on intracellular ATP, which is mainly produced by mitochondrial oxidative phosphorylation. Abnormalities in the function and balance of these cation channels cause disturbances in myocardial excitation and contraction. An insufficient ATP supply and deterioration of ion concentration gradients during myocardial ischemia results in decreased contractile function and arrhythmic activities.

Protection of the myocardium from ischemia–reperfusion damage has been reported with drugs that modify cation homeostasis such as Ca^2+^ channel blockers [2,3], β-blockers [4], and K^+^ channel openers [5]. A common feature of these agents appears to be cardiosuppression; reduced heart rate and force of contraction results in reduced oxygen requirement and preservation of high-energy phosphonucleotides and protects the myocardium from irreversible damage. Using a coronary-perfused guinea pig ventricular free wall preparation, we have reported that Ca^2+^ antagonists, β-blockers, and K^+^ channel openers show cardioprotective effects against experimental ischemia [6,7]. Although the recovery of contractile force after reperfusion was enhanced by these drugs, the decline in contractile force during ischemia was accelerated. Thus, the cardioprotective effects of these drugs were indeed accompanied by cardiosuppression.

Several types of Cl^−^ channels and transporters have been detected in the sarcolemma and intracellular organelles of cardiomyocytes [8,9,10]. A sarcolemmal cAMP-activated Cl^−^ current has been described [8,11] and the corresponding channel protein was revealed to be a cardiac isoform of the cystic fibrosis transmembrane regulator (CFTR) chloride channel [12]. The concentration of Cl^−^ in myocardial cells is estimated to be 10–30 mM and the calculated equilibrium potential for Cl^−^ is around −50 to −70 mV; the flow of Cl^−^ would result in inward currents (Cl^−^ efflux) at normal resting membrane potentials and outward currents (Cl^−^ influx) on depolarization. However, the impact of a Cl^−^ channel current on the myocardial action potential is limited under normal conditions, probably because of the dominance of cation currents. Besides action potential formation, experimental evidence has suggested that Cl^−^ channels and transporters may be involved in the regulation of a large repertoire of cellular functions, including cellular excitability, intracellular organelle acidification, and cell volume homeostasis [8,9,10]. Attenuation of arrhythmia and intracellular acidosis during ischemia and reperfusion was reported with interventions that modify chloride ion homeostasis [13,14,15].

In our previous study, we applied chloride blockers, anthracene-9-carboxylic acid (9-AC), and 4-acetamide-4′-isothiocyanato-stilbene-2,2′-disulfonic acid (SITS) to our coronary perfused ventricular ischemia–reperfusion model and found that they enhanced the recovery of contractile force after reperfusion without decreasing the contractile force under normal conditions [16]. Although such a pharmacological profile was attractive, their cytotoxicity may have hampered their therapeutic application [17,18]. PAK-200 (2-[benzyl(phenyl)amino]ethyl 1,4-dihydro-2,6-dimethyl-5-(5,5-dimethyl-2-oxo-1,3,2-dioxaphosphorina n-2-yl)-1-(2-morpholinoethyl)-4-(3-nitrophenyl)-3-pyridinecarboxylate) is a compound with a dihydropyridine-related structure similar to that of nifedipine [19]. PAK-200 was reported to interact with the P-glycoprotein and reverse drug resistance in several cell lines [19,20,21]. Since the P-glycoprotein and the cAMP-activated Cl^−^-channel bear structural homology and belong to the same superfamily of ATP-binding cassette transporters [22], it is possible that PAK-200 blocks the Cl^−^ channel and has cardioprotective effects. In the present study, we report that PAK-200 indeed blocks the Cl^−^ channel in cardiomyocytes and that it has cardioprotective effects against myocardial ischemia–reperfusion injury.

## 2. Materials and Methods

Guinea pigs weighing 300 to 400 g of both sex were anesthetized with isoflurane and the hearts were isolated and perfused via the aorta with Tyrode’s solutions (gassed with 100% O_2_ and warmed to 36 °C) of the following composition (mM concentration): NaCl 143, KCl 4, MgCl_2_ 0.5, CaCl_2_ 1.8, NaH_2_PO_4_ 0.33, glucose 5.5, and HEPES 5 (pH 7.3). After the blood was washed out, the heart was perfused successively with nominally Ca^2+^-free Tyrode’s solution for 10 min and the same solution containing 0.6 mg/mL collagenase (Yakult, Tokyo, Japan) for about 10 min. Thereafter, the collagenase was washed out by 100 mL of the KB solution (cell storage solution by Isenberg and Klockner [23]) of the following composition (mM concentration): glutamic acid 70, taurine 15, KCl 30, KH_2_PO_4_ 10, MgCl_2_ 0.5, glucose 11, HEPES 10, and EGTA 0.5 (pH 7.3). The ventricular cells were stored in KB solution until used. Effects of PAK-200 on the membrane currents were examined by voltage-clamp experiments under the whole-cell configuration. For measurement of peak-inward and steady-state currents, the glass pipettes contained the following solution (mM concentration): K^+^ aspartate 70, KCl 40, HEPES 5, MgCl_2_ 1, K_2_ATP 5, EGTA 10, and Na^+^_2_ creatine phosphate 5 (pH 7.2). The extracellular solution was Tyrode’s solution. For measurement of the chloride currents, the glass pipettes contained the following solution (mM concentration): Cs^+^ aspartate 70, CsCl 30, HEPES 5, MgCl_2_ 1, Mg^2+^ATP 5, EGTA 5, and tetraethylammonium chloride 20 (pH 7.2). The extracellular solution was the following composition (mM concentration): NaCl 143, CsCl 5.4, MgCl_2_ 0.5, CaCl_2_ 2.5, CdCl_2_ 0.1, BaCl_2_ 1, glucose 11, and HEPES 5.5 (pH 7.4). The amplifier used was Axopatch 200B (Molecular Devices, San Jose, CA, USA). Data acquisition and analysis were performed with Pclamp software (version 10.5; Axon Instruments, Foster City, CA, USA).

The contractile force of the right atria was isometrically measured with a force-displacement transducer TB-611T (Nihon Kohden Corporation, Tokyo, Japan), which was connected to a carrier amplifier AP-621G (Nihon Kohden Corporation, Tokyo, Japan). The analog output signal of the amplifier was digitized by an analog-to-digital converter, Power Lab/4SP, and the beating rate was analyzed with computer software Chart 7 (ADInstruments, Bella Vista, Australia).

Coronary-perfused Langendorff hearts were made from Hartley strain guinea pigs of both sexes weighing 350 to 400 g. After the atria were cut away and the left coronary artery was sutured, the left ventricle and septum were also cut away. The right ventricular wall was perfused at a constant flow rate of 2.0 mL/min at 37 °C with a physiological salt solution of the following composition gassed with 95% O_2_-5% CO_2_ (mM concentration): NaCl 135, KCl 5, CaCl_2_ 2, MgCl_2_ 1, NaHCO_3_ 15, and glucose 5.5 (pH 7.4). The base of the wall was pinned to the bottom of an organ bath (endocardial surface down) and its apex was attached to a force-displacement transducer TB-611T (Nihon Kohden Corporation, Tokyo, Japan). Electrical field stimulation of 3 ms duration was applied through bipolar platinum electrodes at a frequency of 2 Hz (about 1.5× threshold strength). The resting tension on each preparation was applied so that the muscle was stretched to the peak of its length–tension curve. All preparations were allowed to equilibrate for at least 1.5 h before the drugs were applied. No-flow ischemia was produced by stopping the coronary perfusion for 30 min, which was followed by reperfusion for 60 min. During the no-flow ischemia, the organ bath was gassed with 95% N_2_-5% CO_2_ to minimize O_2_ reaching the preparation. PAK-200 (1 μM) or vehicle (0.01% dimethylsulfoxide) was applied through the cannula 30 min prior to no flow ischemia (30 min) and washed out on reperfusion.

Action potentials were recorded by standard microelectrode penetrations into the endocardial surface of the coronary-perfused ventricular preparations. The glass microelectrodes filled with 3 M KCl had resistances of 20 to 30 Mohms. The output of a microelectrode amplifier (Nihon Kohden, MEZ8201) with high input impedance and capacity neutralization was digitized by an analog-to-digital converter Power Lab/4SP and analyzed with computer software Chart 7 (ADInstruments, Bella Vista, Australia). The parameters measured were the resting membrane potential (RP), overshoot (OS), maximum rate of rise (dV/dt_max_), and action potential duration at 90% repolarization (APD_90_).

The coronary-perfused ventricular preparations were quickly freeze-clamped with aluminum tongs and kept in liquid nitrogen under normoxic conditions after 30 min of ischemia and after 60 min of reperfusion. The frozen muscles were then pulverized and homogenized. Determination of ATP content was performed with the firefly luminescence method as previously described [24]. 

For the fluorescence measurements, cells were plated on coverslips attached to an experimental chamber placed on a fluorescence microscope (Olympus IX70). Data acquisition and analysis were performed with Aquacosmos software (version 2.5; Hamamatsu Photonics, Hamamatsu, Japan). Mitochondrial membrane potential was evaluated using the potentiometric indicator tetramethylrhodamine, ethyl ester (TMRE). The cells were loaded with TMRE for 30 min at 37 °C. The cells were excited at 543 nm from a Xenon lamp and the emission was detected in the range of 580 to 600 nm.

Cytoplasmic Ca^2+^ was monitored with the fluorescent probe Fura 2. The cells were loaded with the 5 μM Fura 2-AM for 30 min at 37 °C. They were excited at 340 and 380 nm and the emission (>500 nm) was separated with a dichroic mirror. Calibration was performed in situ. After loading the cell with Fura 2-AM, the extracellular solution was changed to the Ca^2+^-free Tyrode’s solution containing 10 mM EGTA, 5 μM FCCP (carbonyl cyanide p-trifluoromethoxyphenylhydrazone), 5 μM rotenone, and 10 μM ionomycin. After 10 to 20 min, the fluorescence ratio reached minimum values (R_min_). Then, the extracellular solution was changed to normal Tyrode’s solution containing 5 μM FCCP, 5 μM rotenone, and 10 μM ionomycin. After 1 to 2 min, the fluorescence ratio reached maximum values (R_max_). Intracellular Ca^2+^ concentration corresponding to the measured ratio values was calculated as described earlier [25].

Mitochondrial Ca^2+^ was measured with the positively charged fluorescent probe, Rhod 2. Myocytes were loaded with 10 μM Rhod 2-AM for 120 min at 4°C and then incubated for 30 min at 37 °C. This two-step cold loading/warm incubation protocol achieves exclusive loading of Rhod 2 into the mitochondria [26]. The cells were excited at 543 nm and emission was detected in the range of 580 to 600 nm. 

PAK-200 was generously supplied by Nissan Chemical Corporation (Tokyo, Japan). The compound was dissolved in dimethyl sulfoxide (final concentration 0.01%). Fluorescent probes were obtained from Dojindo Laboratories (Kumamoto, Japan). All other chemicals were of the highest commercially available quality.

Data were presented as means ± standard error of the mean (S.E.M.). The significance of the difference between means was evaluated by the Student’s *t*-test. A *p*-value less than 0.05 was considered statistically significant.

## 3. Results

### 3.1. Effect of PAK-200 on the Peak Inward, Steady-State, and Cl^−^ Currents

**Figure 1:**
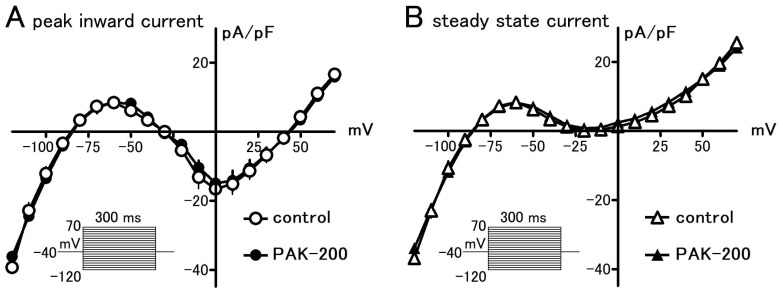
Effect of PAK-200 on peak inward and steady-state currents in isolated guinea pig ventricular cells. Current–voltage relationships for the peak inward (**A**) and steady state (**B**) currents elicited by 300 ms voltage clamp pulses from a holding potential of −40 mV in the absence (open symbols) and presence of 1 μM PAK-200 (closed symbols). Each point with vertical bars represents the mean ± S.E.M. of three experiments.

**Figure 2:**
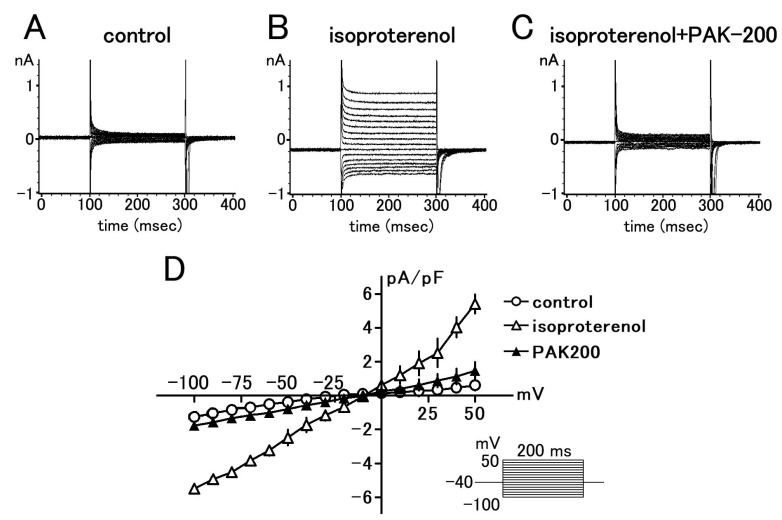
Effect of PAK-200 on the isoprenaline-induced Cl^−^ current in isolated guinea pig ventricular cells. (**A**–**C**): Typical traces of membrane currents elicited by 200 ms voltage clamp pulses from a holding potential of −40 mV to test potentials from −100 mV to +50 mV. (**D**): Current–voltage relationship under control condition (circles) in the presence of 1 μM isoprenaline (open triangles) and in the presence of 1 μM isoprenaline and 1 μM PAK-200 (closed triangles). Each point with vertical bars represents the mean ± S.E.M. of four experiments.

In whole-cell voltage-clamped ventricular myocytes, 1 μM PAK-200 had no significant effect on the peak inward and steady-state membrane currents elicited from a holding potential of −40 mV (Figure 1). Isoprenaline (1 μM) induced a time-independent Cl^−^ current which had an almost linear voltage dependence. PAK-200 markedly blocked this current at all membrane potentials examined (Figure 2); PAK-200, at 1 μM, decreased the isoprenaline-induced current at +50 mV to 27.2 ± 8.1% of that in the absence of the drug.

### 3.2. Effect of PAK-200 on the Spontaneous Beating of the Right Atria

**Figure 3:**
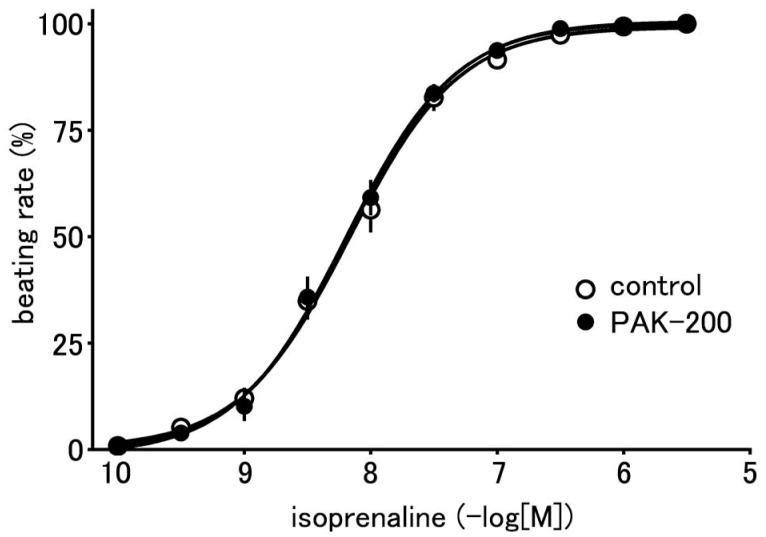
Effect of PAK-200 on the isoprenaline-induced positive chronotropy in the right atria. Concentration–response relationship in the absence (open circles) and presence of 1 μM PAK-200 (closed circles). Each point with vertical bars represents the mean ± S.E.M. of five to seven experiments.

PAK-200 had no effect on the spontaneous beating of the isolated right atria; the spontaneous beating rate before and after the application of 1 μM PAK-200 was 199.6 ± 4.7 and 192.4 ± 4.2 beats per minute (*n* = 5), respectively. PAK-200 had no effect on the chronotropic response to isoprenaline (Figure 3); the pD_2_ values for isoprenaline in the absence and presence of 1 μM PAK-200 were 8.22 ± 0.08 and 8.21 ± 0.08 (*n* = 5–7), respectively.

### 3.3. Mechanical Parameters during Ischemia–Reperfusion and the Effect of PAK-200

**Figure 4:**
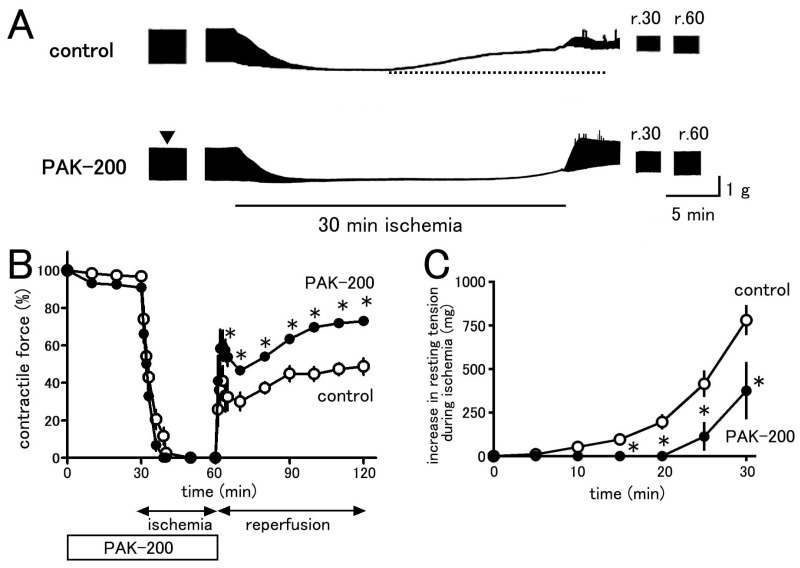
Effects of PAK-200 on the force of contraction of coronary-perfused right ventricular preparations. (**A**): Typical traces obtained in control and PAK-200-treated preparations. The arrowhead indicates the application of 1 μM PAK-200. r.30 and r.60 indicate 30 min and 60 min after reperfusion, respectively. (**B**): Summarized results for contractile force in control (open circles) and PAK-200 treated (closed circles) preparations. (**C**): Summarized results for resting tension in control (open circles) and PAK-200-treated (closed circles) preparations. Each point with vertical bars in panels B and C represents the mean ± S.E.M. of six experiments. Asterisks indicate significant differences (*p* < 0.05) from the corresponding control values.

In coronary-perfused ventricular preparations, experimental no-flow ischemia induced a marked decrease in contractile force and a gradual rise of the resting tension (Figure 4); the contractile force was completely lost within 10 min. On reperfusion, the contractile force recovered only to 48.8 ± 4.4% of pre-ischemic values (*n* = 6). PAK-200 (1 μM) had no effect on the mechanical parameters of the preparations when applied under normal conditions. No difference in contractile force and resting tension was observed between the control and PAK-200-treated preparations. PAK-200 had no effect on the decline of contractile force during ischemia but significantly reduced the rise in resting tension. In preparations pretreated with PAK-200, the contractile force recovered after reperfusion to 73.0 ± 1.7% of pre-ischemic values (*n* = 6).

### 3.4. Electrophysiological Parameters during Ischemia–Reperfusion and the Effect of PAK-200

**Figure 5:**
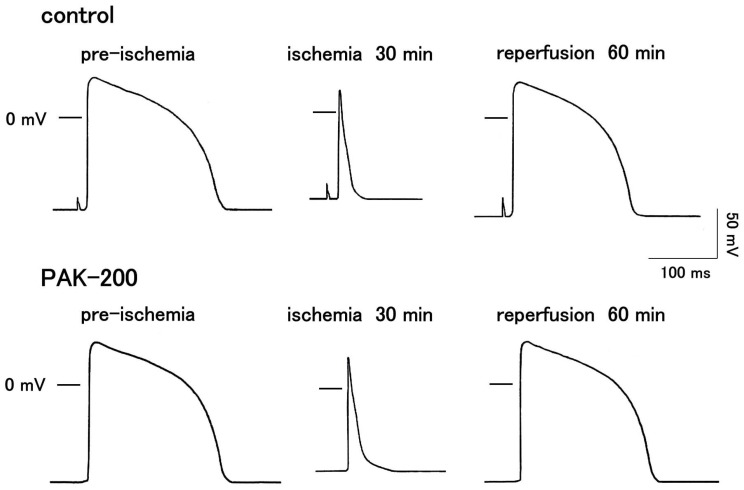
Effect of PAK-200 on the action potential waveform of coronary-perfused right ventricular preparations. Typical traces in control and PAK-200-treated preparations before ischemia, after 30 min of ischemia, and after 60 min of reperfusion.

**Figure 6:**
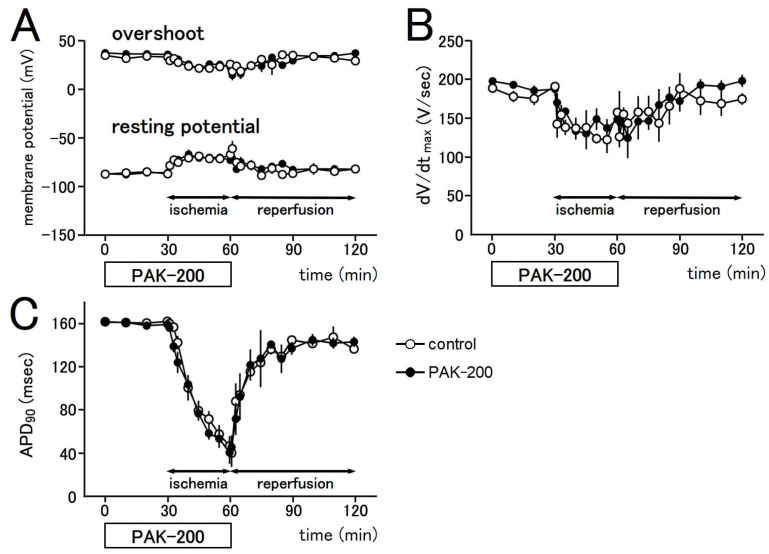
Effect of PAK-200 on the action potential parameters of coronary-perfused right ventricular preparations. Summarized results for the overshoot and the resting potential (**A**), the maximum rate of rise (**B**), and the action potential duration at 90% repolarization (**C**) in control (open circles) and PAK-200-treated (closed circles) preparations. Each point with vertical bars represents the mean ± S.E.M. of six experiments.

The action potential waveform of the coronary-perfused ventricular preparations markedly changed during ischemia but mostly recovered after reperfusion (Figure 5 and Figure 6). The resting potential (RP) decreased (became less negative) by 10% during the first minute of ischemia. A 20 to 30% decrease in overshoot (OS) and the maximum rate of rise (dV/dt_max_) were also observed. These parameters remained relatively unchanged during the rest of the ischemic period. A gradual but significant decrease in the action potential duration proceeded during the ischemic period, which recovered close to preischemic values after reperfusion. The action potential duration at 90% repolarization (APD_90_) decreased to 32.0 ± 5.4% of preischemic values at 30 min after the start of ischemia and recovered to 84.5 ± 2.9% at 60 min after reperfusion (*n* = 6). PAK-200 (1 μM) had no effect on the electrophysiological parameters of the preparations under normal conditions and during ischemia (*n* = 6).

### 3.5. Arrhythmic Contractions after Reperfusion and the Effect of PAK-200

**Figure 7:**
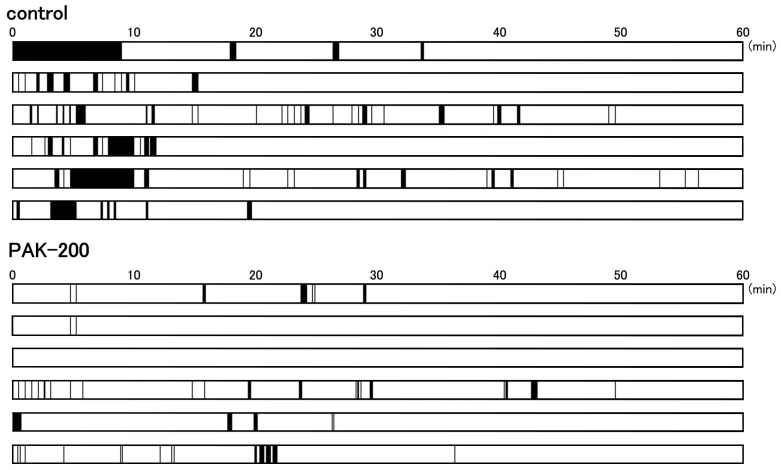
Effect of PAK-200 on arrhythmic contraction after reperfusion in coronary-perfused right ventricular preparations. The horizontal columns represent each preparation and the black area indicates the occurrence of arrhythmic contractions.

Arrhythmic contractions were observed in all of the coronary-perfused ventricular preparations after reperfusion (Figure 4A and Figure 7). The incidence of arrhythmic contractions tended to be higher during the first 10 min after reperfusion. PAK-200 markedly decreased the incidence of arrhythmias after reperfusion.

### 3.6. Tissue ATP Level during Ischemia–Reperfusion and the Effect of PAK-200

**Figure 8:**
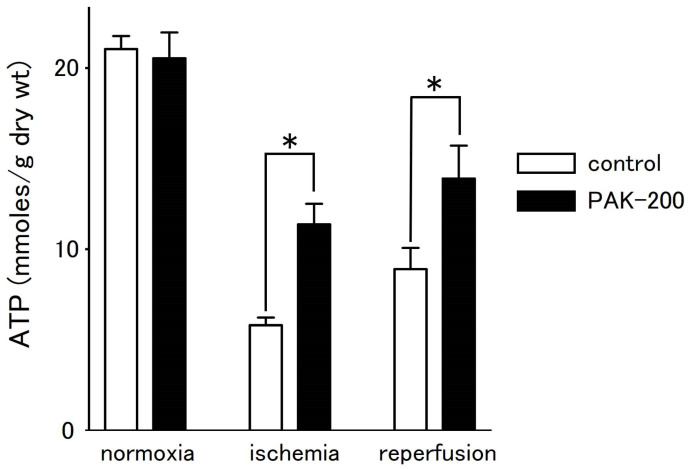
Effect of PAK-200 on the ATP content of coronary-perfused right ventricular preparations. Summarized data from the control (open columns) and PAK-200 treated (closed columns) preparations before ischemia, after 30 min of ischemia, and at 60 min after reperfusion. Each column with vertical bars represents the mean ± S.E.M. of five experiments. Asterisks indicate significant differences (*p* < 0.05) from the corresponding control values.

In tissues, the ATP content of the coronary-perfused ventricular preparations decreased during ischemia and partially recovered after reperfusion (Figure 8). Tissue ATP level after 30 min of ischemia and after 60 min of reperfusion was 28.3 ± 2.1% and 43.2 ± 7.0% of that before ischemia, respectively (*n* = 5). PAK-200 had no effect on tissue ATP content under normal conditions but attenuated the decrease during ischemia. The ATP content of PAK-200-treated preparations was about twice that of control values after 30 min of ischemia; the ATP level of PAK-200-treated preparations after 30 min of ischemia and after 60 min of reperfusion was 56.6 ± 7.1% and 70.0 ± 12.3% of that before ischemia, respectively (*n* = 5).

### 3.7. Effect of PAK-200 on Mitochondrial Membrane Potential

**Figure 9:**
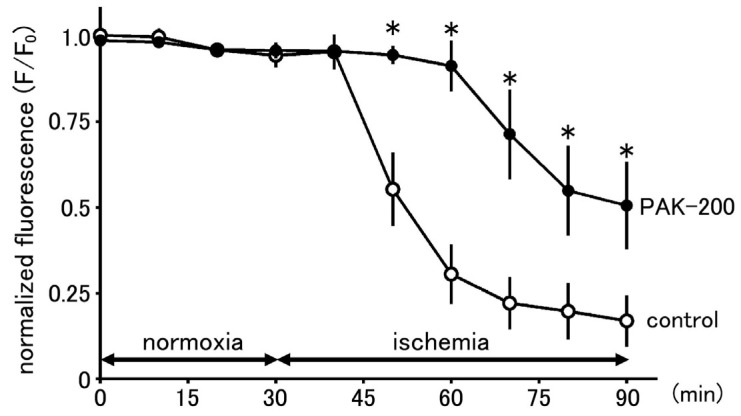
Effect of PAK-200 on the mitochondrial membrane potential during exposure to ischemia-mimetic extracellular solution. The mitochondrial membrane potential of TMRE-loaded cardiomyocytes untreated (control; open circles) or treated (closed circles) with 1 μM PAK-200 was expressed as fluorescence intensity normalized by the initial value. Each point with vertical bars represents the mean ± S.E.M. of 13–14 cells. Asterisks indicate significant differences (*p* < 0.05) from the corresponding values in untreated cardiomyocytes.

The effect of PAK-200 on mitochondrial membrane potential was assessed with TMRE-loaded ventricular myocytes (Figure 9). The TMRE fluorescence intensity gradually decreased during treatment with an ischemia-mimetic solution, which reflects the depolarization of the mitochondria. TMRE fluorescence intensity after 30 min and 60 min of ischemia was 30.6 ± 9% and 16.9 ± 8% of that before ischemia, respectively (*n* = 14). PAK-200 had no effect on TMRE fluorescence under normoxic conditions but significantly delayed the decrease in TMRE fluorescence during experimental ischemia. TMRE fluorescence in PAK-200-treated myocytes before and after 30 min and 60 min of ischemia was 91.2 ± 7% and 50.6 ± 13% of that before ischemia, respectively (*n* = 13).

### 3.8. Cytoplasmic Ca^2+^ Concentration during Ischemia and the Effect of PAK-200

**Figure 10:**
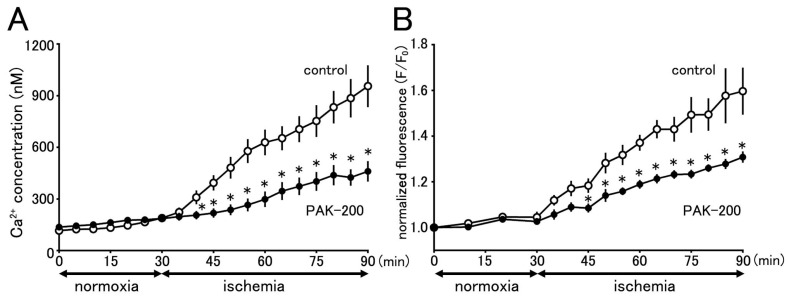
Effect of PAK-200 on intracellular Ca^2+^ concentrations during exposure to ischemia-mimetic extracellular solutions. (**A**): The cytoplasmic Ca^2+^ concentration of Fura-2-loaded cardiomyocytes untreated (control; open circles) or treated with 1 μM PAK-200 (closed circles) was calculated from fluorescence ratios under excitation at 340 and 380 nm. Each point with vertical bars represents the mean ± S.E.M. of 10–15 cells. (**B**): The mitochondrial Ca^2+^ concentration of Rhod-2 treated cardiomyocytes untreated (control; open circles) or treated with 1 μM PAK-200 (closed circles) was expressed as fluorescence intensity normalized by the initial value. Each point with vertical bars represents the mean ± S.E.M of 9–11 cells. Asterisks in both panels indicate significant differences (*p* < 0.05) from the corresponding values in untreated cells.

The changes in cytoplasmic Ca^2+^ concentration during ischemia were assessed with Fura 2-loaded ventricular myocytes (Figure 10A). The Fura 2 fluorescence ratio gradually increased during treatment with the ischemia-mimetic solution, which reflects an increase in cytoplasmic Ca^2+^ concentrations. The cytoplasmic Ca^2+^ concentration calculated from the Fura 2 fluorescence ratio before and after 30 min and 60 min of ischemia was 116.1 ± 15.6 nM, 628.8 ± 74.9 nM, and 955.5 ± 121.7 nM, respectively (*n* = 10). PAK-200 had no effect on Fura 2 fluorescence under normoxic conditions but significantly reduced the increase in fluorescence during experimental ischemia. The calculated cytoplasmic Ca^2+^ concentration in PAK-200-treated myocytes before and after 30 min and 60 min of ischemia was 136.7 ± 12.2 nM, 298.4 ± 45.2 nM, and 460.7 ± 59.5 nM, respectively (*n* = 15).

### 3.9. Effect of PAK-200 on the Mitochondrial Ca^2+^ Concentration

The effect of PAK-200 on mitochondrial Ca^2+^ concentration during ischemia was assessed with Rhod 2-loaded ventricular myocytes (Figure 10B). The Rhod 2 fluorescence intensity gradually increased during treatment with the ischemia-mimetic solution, which reflects an increase in mitochondrial Ca^2+^ concentration. The Rhod 2 fluorescence intensity after 30 min and 60 min of ischemia was 137.1 ± 3.5% and 159.7 ± 10.3% of the value before ischemia, respectively (*n* = 9). PAK-200 had no effect on Rhod 2 fluorescence under normoxic conditions but significantly reduced the increase in fluorescence during experimental ischemia. The Rhod 2 fluorescence in PAK-200-treated myocytes after 30 min and 60 min of ischemia was 118.9 ± 1.9% and 130.8 ± 2.5% of the value before ischemia, respectively (*n* = 11).

## 4. Discussion

The present study was undertaken to clarify the cardioprotective effects of PAK-200 against experimental ischemia and reperfusion. PAK-200 is a dihydropyridine analog that was reported to sensitize multidrug-resistant tumor cells [19,20,21]. In the present study, PAK-200 was found to inhibit the isoprenaline-induced Cl^−^ current in cardiomyocytes (Figure 2). This is not surprising because P-glycoprotein and the cAMP-activated Cl^−^ channel are considered to bear structural homology and belong to the same superfamily of molecules [8]. Although the chemical structure of PAK-200 partially resembles that of nifedipine [19], it had no effect on the peak-inward current and steady-state currents indicating a lack of effect on Ca^2+^ and K^+^ currents (Figure 1). It did not affect the ventricular action potential parameters including the maximum rate of rise (Figure 5 and Figure 6), which correlates with the Na^+^ current. In addition, PAK-200 had no effect on the contractile force during normoxia (Figure 4), which indicated that PAK-200 did not affect CICR from the SR. In the spontaneously beating right atrium, PAK-200 did not affect the beating rate, which suggests that it does not affect the ionic currents involved in the pacemaker depolarization of the sinus node such as Ca^2+^ current, K^+^ current, and the hyperpolarization-activated current (I_f_) [27,28]. PAK-200 had no effect on the chronotropic response to isoprenaline (Figure 3), which indicated the lack of effect of PAK-200 on β-adrenergic receptors. Thus, PAK-200 appears to be a Cl^−^-channel blocker with no effect on major cationic currents. Whether PAK-200 affects other Cl^−^ currents, such as the Ca^2+^-activated Cl^−^ current and the swelling-activated Cl^−^ current, remains to be clarified.

The coronary-perfused myocardial ischemia–reperfusion model in the present study was the same as that used in our previous studies [6,7,16,29]. The marked decrease in contractile force during ischemia (Figure 4) could be explained by multiple factors including shortening of action potential duration (APD), decreased Ca^2+^ release from the sarcoplasmic reticulum, and intracellular acidosis [6,30,31]. During the last 10 min of the ischemic period, a gradual rise in resting tension (contracture) was observed. Such a phenomenon could be attributed to an increase in cytoplasmic Ca^2+^ concentration and rigor shortening of the myofilaments [32,33]. Concerning the action potential parameters, decreases in resting potential (RP), maximum rate of rise (dV/dt_max_), and overshoot (OS) were observed during the first 10 min of ischemia (Figure 5 and Figure 6). The primary cause of these changes during ischemia is a decrease in intracellular ATP concentration [34]. The ATP consumption by the three major ATPases, actomyosin-ATPase, sarcoplasmic reticulum Ca^2+^-ATPase (SERCA), and the sarcolemmal Na^+^-K^+^ ATPase, were estimated to be about 70%, 15%, and 9%, respectively, of the total ATP consumption in guinea pig ventricular myocardium [35]. Much of the changes observed during ischemia can be ascribed to decreased activity of these ATPases. A decrease in the actomyosin ATPase activity directly causes a decrease in contractile force. A decrease in SERCA activity will reduce Ca^2+^ sequestration into the SR, which leads to a rise in the basal cytoplasmic Ca^2+^ concentration and muscle contracture. The decrease in intracellular ATP during ischemia results in the opening of the sarcolemmal ATP-sensitive K^+^ channel and a shortening of APD. As the cytoplasmic Ca^2+^ to activate the actomyosin ATPase is provided by the SR through the CICR mechanism, a decreased APD, which means a decrease in the Ca^2+^ to trigger CICR, together with the decrease in SR Ca^2+^ content, will cause a decrease in contractile force [6]. Decreased activity of the Na^+^-K^+^ ATPase during ischemia causes a decrease in the transsarcolemmal concentration gradient of both Na^+^ and K^+^. In addition, the opening of the ATP-sensitive K^+^ channel will allow K^+^ to move across the sarcolemma from the cytoplasm to the extracellular space and aggravate the loss of the transsarcolemmal K^+^ gradient. The resulting decrease in RP produces a reduction in dV/dt_max_ by increasing the steady-state inactivation of the Na^+^ channel [36,37]. 

PAK-200 had no effect on the decrease in contractile force, RP, dV/dt_max_, OS, and APD during ischemia (Figure 4, Figure 5 and Figure 6). As mentioned above, the major factor causing these changes during ischemia is the opening of the ATP-sensitive K^+^ channel. Using the same experimental ischemia–reperfusion model as in the present study, we reported that the application of NIP-121, an ATP-sensitive K^+^ channel opener, during ischemia accelerated the decrease in APD, while glibenclamide slowed it [6]. In the present study, PAK-200 had no effect on the shortening of APD during ischemia, indicating a lack of direct effect on ATP-sensitive K^+^ channels. In our previous study with Cl^−^-channel blockers [16], both 9AC and SITS significantly delayed the shortening of APD. Thus, the pharmacological profile of PAK-200 may be different from these Cl^−^ blockers. The ischemia-induced rise in resting tension was significantly inhibited by PAK-200 (Figure 4). The most probable explanation is that the preservation of ATP level by PAK-200 (Figure 8) resulted in the preservation of the Ca^2+^-handling capacity of the cell and attenuated the rise in cytoplasmic Ca^2+^ concentration and resting tension. As PAK-200 did not enhance the decrease in contractile force, PAK-200 probably does not reduce ATP consumption but rather preserves ATP production. 

On reperfusion, the contractile force and resting tension were partially recovered (Figure 4), which correlated with the partial recovery of the ATP level (Figure 8). The cardioprotective effects of PAK-200 on the contractile force and resting tension as well as on the ATP level were partial, which could be explained by the partial protective effects of PAK-200 on the mitochondria (Figure 9 and Figure 10B). The ATP level only partially recovered even after reperfusion for 60 min which implies that some irreversible damage occurred in the mitochondria (Figure 8). Intermittent arrhythmia was observed after reperfusion in all of the preparations either untreated or treated with PAK-200. PAK-200 tended to reduce the incidence of arrhythmia during early reperfusion (Figure 7). It is probable that this antiarrhythmic effect of PAK-200 is closely related to Cl^−^ homeostasis because similar antiarrhythmic effects have been reported with agents with Cl^−^-blocking effects [14,16]. CFTR overexpression in mice was reported to provide an arrhythmogenic substrate [38]. Thus, the Cl^−^-channel blockade appears to exert antiarrhythmic effects against myocardial ischemia; the accumulation of information is necessary to understand their mechanisms of action. 

To obtain information on the mechanism of action for PAK-200 to preserve ATP production, we examined the intracellular effects of PAK-200 in isolated cardiomyocytes. According to the chemiosmotic theory [39], the mitochondrial membrane potential (Δφ_m_) is the sole energy-transduction intermediate between the respiratory chain and proton-translocating ATP synthetase and, thus, loss of Δφ_m_ indicates loss of mitochondrial ATP synthesis. The time course of mitochondrial depolarization during ischemia was reported to correlate with ATP exhaustion in cardiomyocyte-derived HL-1 cells [40]. In the present study, treatment of the TMRE-loaded cardiomyocytes with an ischemia-mimetic solution induced a gradual loss of Δφ_m_ (Figure 9). The time course of this loss of Δφ_m_ was significantly delayed by PAK-200, indicating that the driving force for ATP production by the ATP synthase was partly preserved. Thus, the attenuation of the ischemia-induced decrease in ATP by PAK-200 could be explained by the preservation of mitochondrial ATP production. 

The Δφ_m_ is largely affected by the cytoplasmic Ca^2+^ concentration. Cytoplasmic Ca^2+^ can be absorbed into the mitochondria through the calcium uniporter on the mitochondrial inner membrane [41,42]; changes in the cytoplasmic Ca^2+^ concentration under physiological and pathological conditions affect the intramitochondrial Ca^2+^ concentration. The rise in mitochondrial Ca^2+^ concentration may either increase or decrease Δφ_m_ [43,44,45,46]. Increases in the mitochondrial Ca^2+^ concentration caused by physiological increases in the cytoplasmic Ca^2+^ concentration, such as that under positive inotropic stimuli, stimulate the metabolism by the TCA cycle and the electron transfer system, and lead to an increase in Δφ_m_ (more negative mitochondrial membrane potential). This enhances mitochondrial ATP production to meet the demand of the myocardium, producing a larger contractile force. On the other hand, excess increases in mitochondrial Ca^2+^ under pathological conditions such as myocardial ischemia cause mitochondrial swelling and dysfunction which results in a decrease in Δφ_m_ and ATP production [47].

In the present study, an increase in mitochondrial Ca^2+^ was observed in cardiomyocytes exposed to the ischemia-mimetic solution, which paralleled the increase in cytoplasmic Ca^2+^ concentration (Figure 10). The increases in cytoplasmic and mitochondrial Ca^2+^ were both significantly reduced by PAK-200, indicating that the compound can reduce Ca^2+^ overload both in the cytoplasm and mitochondria. Although this mitochondria-protective effect of PAK-200 can explain the cardioprotective effects of the compound during experimental ischemia and reperfusion, the mechanism of action of PAK-200 is not clear at present. Among the Ca^2+^ transporters on the mitochondrial inner membrane, the Ca^2+^ uniporter is responsible for the influx of cytoplasmic Ca^2+^ into the mitochondrial matrix and the mitochondrial Na^+^-Ca^2+^ exchanger (NCXL) is responsible for Ca^2+^ efflux [43,44,45,48]. Cl^−^ channels and related transporters are present not only on the sarcolemma but also on the mitochondrial membrane. The voltage-dependent anion channel (VDAC) is known to be closely related to mitochondrial Ca^2+^ and function [49]. Whether PAK-200 affects these transporters remains to be clarified.

**Figure 11:**
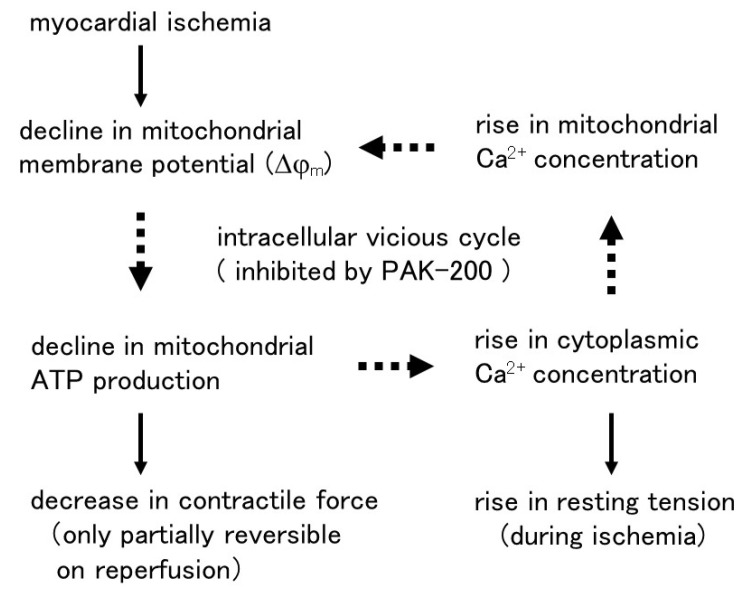
Tentative mechanisms of the cardioprotective effects of PAK-200. PAK-200 inhibits the intracellular vicious cycle during ischemia and enhances the recovery of contractile force after reperfusion.

The results of the present study can be summarized as follows (Figure 11). Insufficient oxygen supply during myocardial ischemia causes an inhibition of mitochondrial electron transport leading to a decline in Δφ_m_. The resulting decrease in mitochondrial ATP production causes not only a loss of contractile activity but also failures in various cellular processes including maintenance of the ion environment; the decrease in the activity of Na^+^-K^+^ ATPase and Ca^2+^ ATPase induces the rise in cytoplasmic Na^+^ and Ca^2+^ concentration. The rise in cytoplasmic Ca^2+^ causes a rise in resting tension during late ischemia and leads to an excessive rise in mitochondrial Ca^2+^ concentration, which causes mitochondrial dysfunction such as a further decrease in Δφ_m_ and ATP production. Thus, these intracellular events during ischemia form a vicious cycle and the cellular condition continuously deteriorates. PAK-200, a dihydropyridine analog that blocks the CFTR Cl^−^ channel, was shown to inhibit this vicious cycle and exert cardioprotective effects to enhance the recovery of contractile force after reperfusion. A characteristic feature of the cardioprotection by PAK-200 was that it was free of cardiosuppression; neither the beating rate nor the contractile force was decreased by PAK-200. Further accumulation of basic information on the role of Cl^−^-related channels and transporters, as well as the mechanism of action of Cl^−^ modulators, would reveal a novel mechanism for cardioprotection. Several dihydropyridine-related compounds have been reported to affect the CFTR Cl^−^ channels [29,50,51] and some of them appeared to have cardioprotective effects similar to those of PAK-200 [29,50]. Re-evaluation of dihydropyridine-related compounds may lead to the discovery of cardioprotective agents acting through novel mechanisms.

## Data Availability

Data are contained within the article.
